# A surgical case of inflammatory abdominal aortic aneurysm that responded remarkably to preoperative steroid therapy

**DOI:** 10.1093/jscr/rjy020

**Published:** 2018-02-20

**Authors:** Makoto Iijima, Ryota Azuma, Tetsuya Hieda, Yutaka Makino

**Affiliations:** Department of Cardiovascular Surgery, Oji General Hospital, Tomakomai, Hokkaido, Japan

## Abstract

We describe the surgical management of a 58-year-old man with inflammatory abdominal aortic aneurysm (IAAA) following treatment with preoperative steroids. The patient was transferred to our institution for abdominal pain and fever. Contrast-enhanced computed tomography showed juxtarenal abdominal aortic aneurysm surrounded by dense perianeurysmal fibrous tissue. Under a diagnosis of IAAA, steroid therapy with prednisolone was initiated to control the perianeurysmal inflammation. It continued for 3 weeks with a decreasing dose schedule, with remarkable decrease in the soft tissue mass. The patient underwent elective surgery 21 days after commencing steroid therapy. During surgery, adjacent organs were adherent to the aneurysmal wall, but fibrotic change to the retroperitoneum was very limited. He recovered uneventfully, and was discharged on postoperative Day 10. Therefore, it can be concluded that preoperative steroid therapy could minimize the operative risk for IAAAs, and improve surgical outcome.

## INTRODUCTION

Inflammatory abdominal aortic aneurysm (IAAA), characterized by marked thickening of the aortic wall with fibrous extension to the adjacent retroperitoneum and rigid adherence of adjacent structures to the aneurysm, is a relatively rare disease that occurs in 3–10% of all abdominal aortic aneurysms (AAAs) [1]. The disease was first described by Walker in 1972, and several similar cases have since been reported [2]. Moreover, perioperative mortality in patients with IAAAs is more than three times as high as that with atherosclerotic AAA in some series, due to the potential for iatrogenic injury in the presence of perianeurysmal adhesions [1, 3]. Some authors insist on the effectiveness of preoperative steroid therapy in the management of IAAA, as a complementary treatment to reduce the mass that has disadvantageous effects on the operative outcome [4]. Herein, we report a case of a symptomatic IAAA that was successfully treated with preoperative steroid therapy and subsequent surgical resection.

## CASE REPORT

A 58-year-old man presented to a local hospital with a 5-day history of abdominal pain and fever. His medical history was unremarkable, with no hypertension or regular medications, but positive for active smoking. Plain computed tomography (CT) showed enlargement of the abdominal aorta and a surrounding soft tissue mass. He was transferred to our institution for further evaluation and treatment.

**Figure 1:**
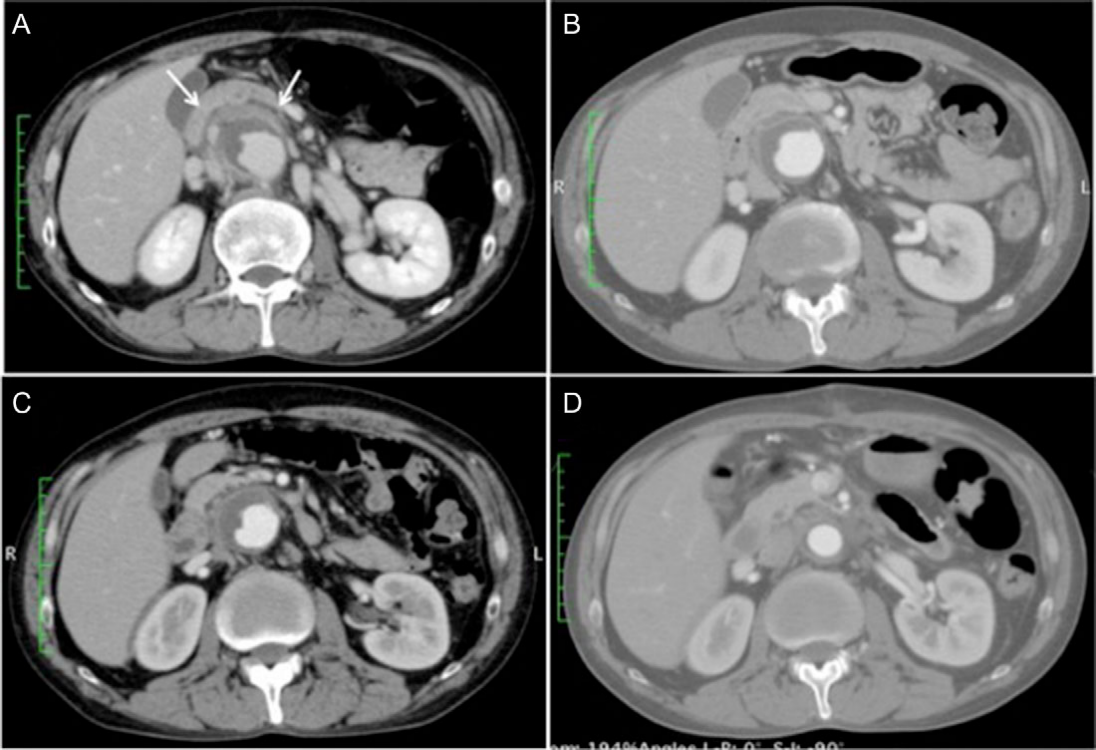
(**A**) Contrast-enhanced computed tomography scan at the time of admission showing a 50-mm juxtarenal abdominal aortic aneurysm surrounded by dense fibrous tissue (white arrows). (**B** and **C**) The perianeurysmal fibrous tissue became smaller along with the course of steroid therapy, 7 and 14 days after the initiation of steroid therapy, respectively. (**D**) Postoperative computed tomography scan showing a patent graft with near disappearance of the fibrous tissue.

Upon admission, the patient’s blood pressure was 135/72 mmHg, and he had low-grade fever at 37.4°C. Blood analyses showed a nearly normal level of white blood cells but elevated C-reactive protein of 20.1 mg/dL. Contrast-enhanced CT of the abdominal vessels showed a juxtarenal AAA (maximum diameter, 50 mm) surrounded by dense perianeurysmal fibrous tissue (Fig. 1A). Despite repeated cultures, the causative microorganism was not identified. The clinical presentation and imaging findings led to the diagnosis of a symptomatic IAAA. To control the perianeurysmal inflammation, steroid therapy with prednisolone was initiated at a dose of 30 mg/day, which was gradually reduced to 10 mg/day using inflammatory signs and change in the size of retroperitoneal mass as a reference (Fig. 1B and C). He responded well to steroids initially and his abdominal pain resolved. The patient underwent elective surgery 21 days after commencing steroid therapy.

**Figure 2:**
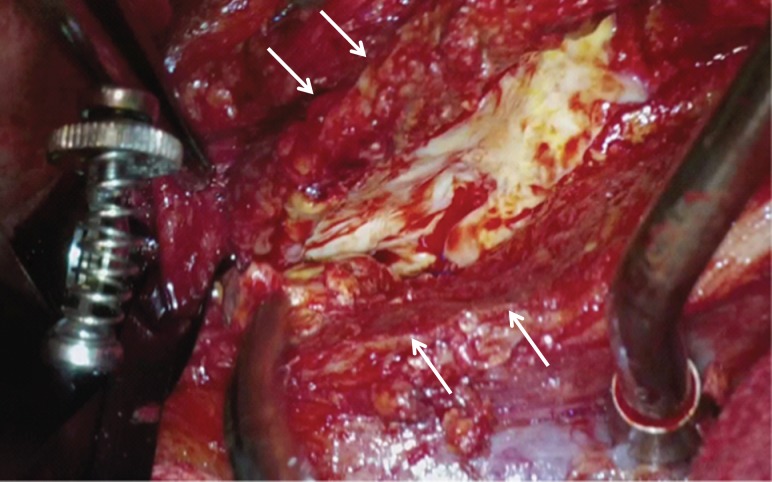
Intraoperative image showing that a small range of the aneurysmal wall was severely thickened (white arrows) but the fibrotic change of the retroperitoneum was limited.

A midline laparotomy exposed the white-changed retroperitoneum. Adjacent organs, including the duodenum and the ileum, were adherent to the aneurysmal wall, but fibrotic change to the retroperitoneum was very limited. After proximal control to clamp suprarenal aorta and distal control to clamp bilateral common iliac artery were obtained in sites distant from the thickened parts of the juxtarenal AAA, the aneurysm was opened longitudinally. The anterior and lateral walls of the aneurysm were significantly thickened (Fig. 2). A Dacron bifurcated graft [Hemashield Gold 14 × 8 mm^2^, MAQUET Cardiovascular LLC, San Jose, CA, USA] was anastomosed proximally to the infrarenal aorta and distally to the bilateral common iliac artery. This operation was performed in 197 min without transfusion.

**Figure 3:**
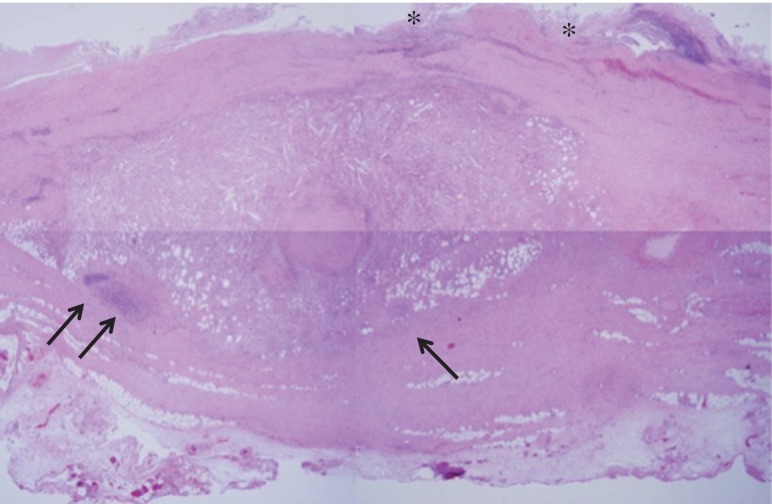
Light microgram of the hematoxylin and eosin-stained aortic wall revealing the collapsed structure of the tunica intima (single asterisk), and the marked thickening in the tunica adventitia due to infiltration by inflammatory cells (black arrows) accompanied with fibrous proliferation.

He recovered uneventfully, and postoperative contrast-enhanced CT revealed a patent graft and a decrease in the retroperitoneal mass (Fig. 1D). Histopathologic examination demonstrated excessively thickened tunica adventitia with infiltration of inflammatory cells such as lymphocytes and fibrosis (Fig. 3). The patient was discharged without subjective symptoms on postoperative Day 10.

## DISCUSSION

IAAAs are a variant of aortic aneurysms characterized by massive thickening of the aneurysmal wall and extensive fibrous adhesion to adjacent tissues and structures. Although rare, their operative mortality is higher than that for atherosclerotic aortic aneurysms, and optimal perioperative management consisting of preoperative medication and subsequent open surgery or endovascular repair is required.

In their reviews, Walker *et al*. [2] identified three characteristics consistently associated with IAAA: (i) abdominal/lower back pain, (ii) anorexia/weight loss and (iii) increased CRP level and/or erythrocyte sedimentation rate. Patients with IAAA usually present at a younger age than those with atherosclerotic aortic aneurysm, and male sex and smoking are two major risk factors for IAAAs [5]. Biochemical findings are useful but not diagnostic. Several case reports recommended the use of CT to diagnose IAAAs, as used in this case. Iino et al. reported a sensitivity of 83.3%, a specificity of 99.7%, and an accuracy of 93.7% for the diagnosis of IAAAs using CT [6].

IAAAs are clearly distinguished from atherosclerotic AAAs by adhesion between aneurysmal wall and adjacent structures such as the duodenum, ileum, ureter and inferior vena cava. Preoperative steroid therapy is particularly effective in the surgical management of cases with IAAAs in which preoperative CT scanning demonstrates the aneurysm and the extremely thickened aortic wall with periaortic inflammation and fibrosis, the so-called ‘mantle-sign’, which makes the operation very challenging due to difficult adhesiotomy [4]. In the present case, steroid therapy played a role in the regression of perianeurysmal fibrous tissue caused by inflammation, allowing us to safely perform open surgery.

An unsolved issue in the preoperative management for IAAAs is the absence of guidelines referring to steroid therapy, especially indication and initial dose. Steroids have a definite anti-inflammatory effect, which might be also implicated in inducing weakening of the aneurysmal wall, as mentioned by Sasaki *et al*. [4]. Preoperative steroid therapy should be administered cautiously in patients with larger aneurysms considering the risk of aneurysmal enlargement. After initiating steroid therapy, periodic imaging studies should be performed to assess the aneurysmal diameter and the surrounding fibrous tissue. We determined the initial dose (0.5 mg/kg/day) by referring to previous literature, all of which were merely case reports dealing with small numbers of patients [7]. Further studies involving more patients are needed to establish the appropriate steroid dose in the management of IAAAs.

Treatment of IAAAs via endovascular repair has become increasingly common in recent years. Compared to open repair, it is less invasive, and the risk of iatrogenic injury in the presence of perianeurysmal adhesions is low. However, the patient’s anatomy was unsuitable for endovascular treatment due to a short neck in this case. We suggest that preoperative steroid therapy is particularly useful when open surgery is the only option, as in the current case.
